# Remarkable cell recovery from cerebral ischemia in rats using an adaptive escalator-based rehabilitation mechanism

**DOI:** 10.1371/journal.pone.0223820

**Published:** 2019-10-11

**Authors:** Chi-Chun Chen, Yu-Lin Wang, Ching-Ping Chang

**Affiliations:** 1 Department of Electronic Engineering, National Chin-Yi University of Technology, Taichung, Taiwan; 2 Department of Biomedical Engineering, National Cheng Kung University, Tainan, Taiwan; 3 Center of General Education, Southern Taiwan University of Science and Technology, Tainan, Taiwan; 4 Department of Physical Medicine and Rehabilitation, Chi Mei Medical Center, Tainan, Taiwan; 5 Department of Medical Research, Chi Mei Medical Center, Tainan, Taiwan; University of Warwick, UNITED KINGDOM

## Abstract

Currently, many ischemic stroke patients worldwide suffer from physical and mental impairments, and thus have a low quality of life. However, although rehabilitation is acknowledged as an effective way to recover patients’ health, there does not exist yet an adaptive training platform for animal tests so far. For this sake, this paper aims to develop an adaptive escalator (AE) for rehabilitation of rats with cerebral ischemia. Rats were observed to climb upward spontaneously, and a motor-driven escalator, equipped with a position detection feature and an acceleration/deceleration mechanism, was constructed accordingly as an adaptive training platform. The rehabilitation performance was subsequently rated using an incline test, a rotarod test, the infarction volume, the lesion volume, the number of MAP2 positive cells and the level of cortisol. This paper is presented in 3 parts as follows. Part 1 refers to the escalator mechanism design, part 2 describes the adaptive ladder-climbing rehabilitation mechanism, and part 3 discusses the validation of an ischemic stroke model. As it turned out, a rehabilitated group using this training platform, designated as the AE group, significantly outperformed a control counterpart in terms of a rotarod test. After the sacrifice of the rats, the AE group gave an average infarction volume of (34.36 ± 3.8)%, while the control group gave (66.41 ± 3.1)%, validating the outperformance of the escalator-based rehabilitation platform in a sense. An obvious difference between the presented training platform and conventional counterparts is the platform mechanism, and for the first time in the literature rats can be well and voluntarily rehabilitated at full capacity using an adaptive escalator. Taking into account the physical diversity among rats, the training strength provided was made adaptive as a reliable way to eliminate workout or secondary injury. Accordingly, more convincing arguments can be made using this mental stress-free training platform.

## Introduction

The entire ischemic stroke population grows at a rate of 15 million people per annum [1], and results in a high medical expense of more than US$90 billion [2]. Although the stroke survival rate has gone beyond 50% as medical science advances, ischemic stroke remains a major cause of physical impairment [3], causes neurological disorders, and impairs physical abilities and mental functions, including cognition, learning, memory, and more. Patients have a poor quality of life accordingly. Physical exercise has been acknowledged as an effective way of rehabilitation, and rehabilitation performance for human patients has been validated using animal models, e.g., rats [4–6]. Currently, treadmills and running wheels are the most frequently used training platforms for animals, and satisfactory performance has been observed in stroke prevention [7, 8], but not yet in rehabilitation [9–11]. This is simply because rats, after middle cerebral artery occlusion (MCAo) surgeries, become physically weaker than healthy ones. As a consequence, secondary injuries due to overtraining have been inevitably seen among rehabilitated rats, once they cannot keep up with the running speed of a non-adaptive training platform. Individuals in a rehabilitated group may even underperform those in a non-rehabilitated counterpart [10, 11]. This finding may be true for human patients, and the importance of developing an adaptive rehabilitation platform for human use is highlighted accordingly.

Currently, treadmills and running wheels are commonly employed as animal training platforms for rehabilitation purposes. Robot-assisted rehabilitation is performed using a treadmill [12, 13], while non-assisted counterparts are conducted using either a running wheel or a treadmill [4–6, 9–11]. In a representative study on the robot-assisted rehabilitation issue [14], a rat, when walking for rehabilitation, was suspended using a robotic harness. A satisfactory rehabilitation performance was seen, and this rehabilitation mechanism has been applied to human patients. However, a major disadvantage is that it took a tremendous amount of effort to tune the running speed of a treadmill due to the wide physical diversity among rats, and besides a robotic arm was controlled in a complicated way. In most cases, rats were electrically stimulated at the end of a treadmill, which affects mental health conditions [15], results in negative physiological effects [16, 17] and leads to unconvincing arguments [18, 19]. A major disadvantage of a forced, i.e., motor-driven, running wheel is that rats were observed to fall down and get hurt [20]. Once the rats were unable to catch up with the wheel, they held onto the rail, and were brought to the upper part of the wheel accordingly [21, 22]. In a voluntary running wheel, rats can run of their own free will, meaning that they can take a break when required. Nonetheless, rehabilitation performance is susceptible to the physical conditions of the rats, and rats undergoing rehabilitation must be screened in advance due to high inter-subject variability [23, 24]. In short, there are some disadvantages in today’s commercial training platforms.

More importantly, it must be pointed out that there has long been a controversy over the efficacy of rehabilitation treatment using forced training facilities, that is, treadmills and running wheels, in the literature. For instance, the rehabilitation treatment was found to be effective in [25], while ineffective in [9, 10]. The contradiction was caused perhaps for the following reason. It was suggested in [26] that moderate treadmill activity at 25 m/min would elevate serum corticosterone. Corticosterone has been known as a typical sign of chronic stress, which usually causes a body weight loss and spleen atrophy [16], indicating a response of negative adaptation to stress. Furthermore, corticosterone was shown to reduce BDNF availability in the rat hippocampus [27]. Hence, there is definitely a point to develop an innovative rehabilitation mechanism, such that the level of serum corticosterone can be reduced significantly, and hopefully more convincing argument can be made accordingly to resolve the efficacy controversy.

There are mainly two types of physical training: aerobic and anaerobic (strength) training, and a comparison between both is made as follows. As defined by Vileka et al. in [28], strength training is short bursts of intensive and repetitive training, and was illustrated therein by the following example. A rat was incrementally loaded at its tail with an object ranging between 50 and 100% of the body weight throughout an 8-week training program. Three–five sets of 8–12 repetitions, with a 1-min rest between repetitions and a 2-min rest between sets, were performed for 3 or 4 days per week. As pointed out in [29], strength training program was designed for muscle hypertrophy and neural adaptation improvement. Instead, aerobic training refers to a long-duration training for improvement in oxidative metabolism. For example, all the rats waiting for training were acclimatized to a 9-channel motor-driven treadmill at a speed of 10 m/min for a 10-min duration per day for 1 week [28]. The aerobic training program aimed to elevate VO_2max_ via a stimulus that increases the oxygen-carrying capacity and improves the oxygen utilization efficiency in the body as well. In this work, all the rats, when rehabilitated, were unloaded, and breathed as usual, in long-duration ladder climbing workout. According to the previous definitions, the ladder climbing movement obviously invokes an aerobic mechanism.

Rats have been observed to climb upward as an instinctive behavior, inspiring our team to develop an escalator-based rehabilitation mechanism. Although there have been a number of publications on the issue of ladder climbing-based training mechanisms, e.g. [30–33], it must be stressed here that all the training facilities therein were static ladder, unlike this work. For the first time in the literature, the running speed of an escalator adapted all the time to the physical conditions of rats for rehabilitation purposes. Consequently, the rehabilitation performance can be well improved in a workout injury-free environment, particularly under mental stress-free conditions, as validated in [9, 10].

## Materials and methods

**Fig 1:**
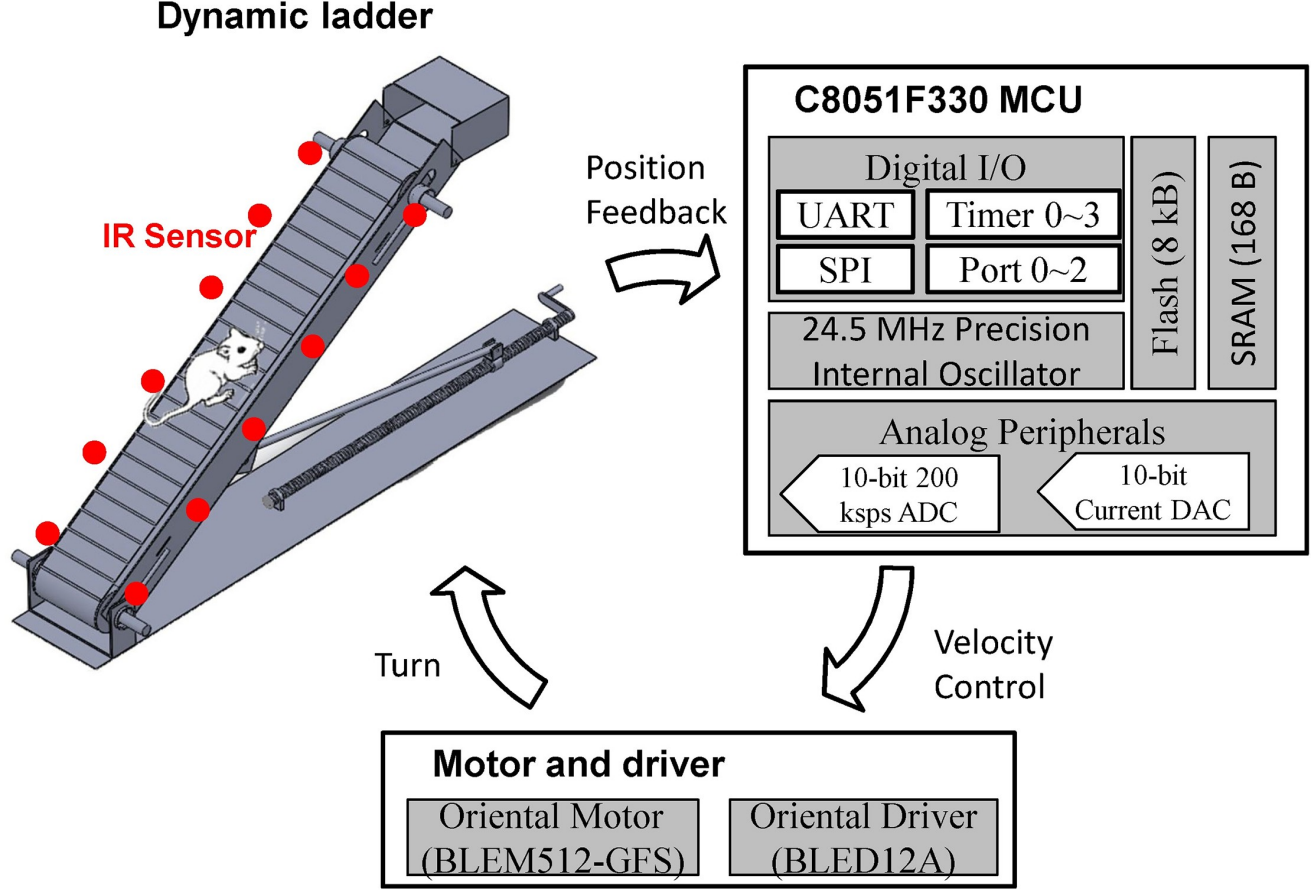
Major components involved in the presented motor-driven escalator.

As illustrated in Fig 1, the adaptive rehabilitation mechanism mainly involved a C8051F330 microcontroller, a motor together with a driver and a dynamic ladder platform. The position, detected by IR sensors, was fed into the microcontroller to tune the running speed of the motor-driven escalator. Adaptive rehabilitation service was provided to patients with diverse physical conditions.

The construction of the presented rehabilitation platform was composed of 5 stages: (i) mechanism design, (ii) circuit design, (iii) running speed control, (iv) performance validation and (v) animal tests. Stages 1–3 were hardware development, stage 4 was for feature tests, e.g., position detection, and stage 5 was to test the patients’ rehabilitation performance using this platform.

### Mechanism design

**Fig 2:**
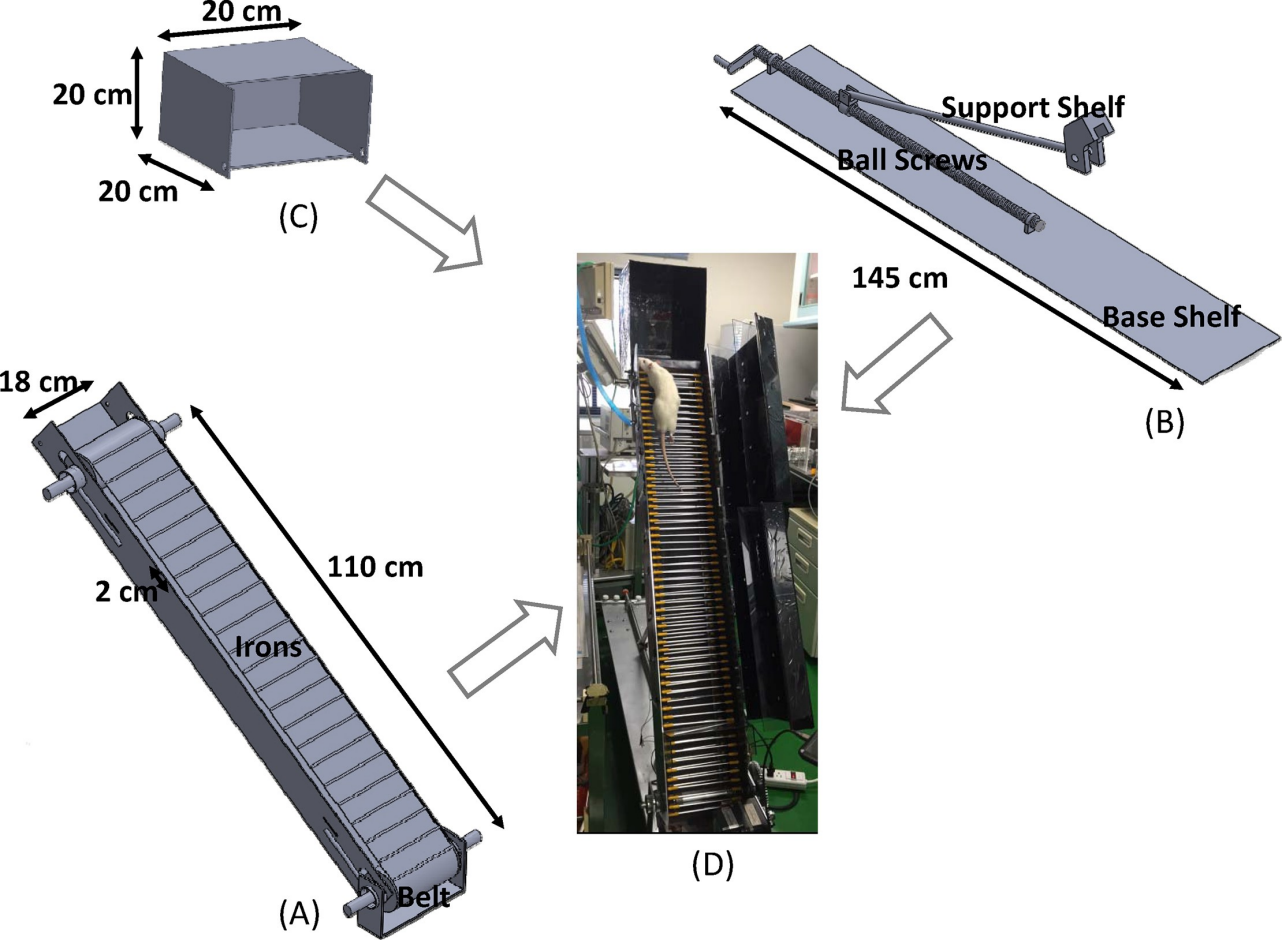
Mechanism design consisting of (A) the main body, (B) an adjustable tilt shelf, (C) an empty cube, and (D) a photo of the mechanical assembly.

The presented AE mechanism comprises a rat escalator as the main body, illustrated in Fig 2A, and an adjustable tilt shelf in Fig 2B and an empty cube in Fig 2C, and the assembled mechanism is presented in Fig 2D. The side belt-driven long-shaped main body measures 110 cm by 18 cm, and has runway rails with a 2 cm pitch. The adjustable tilt shelf is composed of a base shelf, ball screws and a support shelf, providing an inclination angle between 0 and 90° for different levels of strength training. As presented in [30–33], rats are trained at fixed, rather than variable, inclination angles between 25° and 90°. The 20 cm×20 cm×20 cm empty cubic is designed for the purposes of rats’ rest when they reach the top of the escalator.

### Circuit design

**Fig 3:**
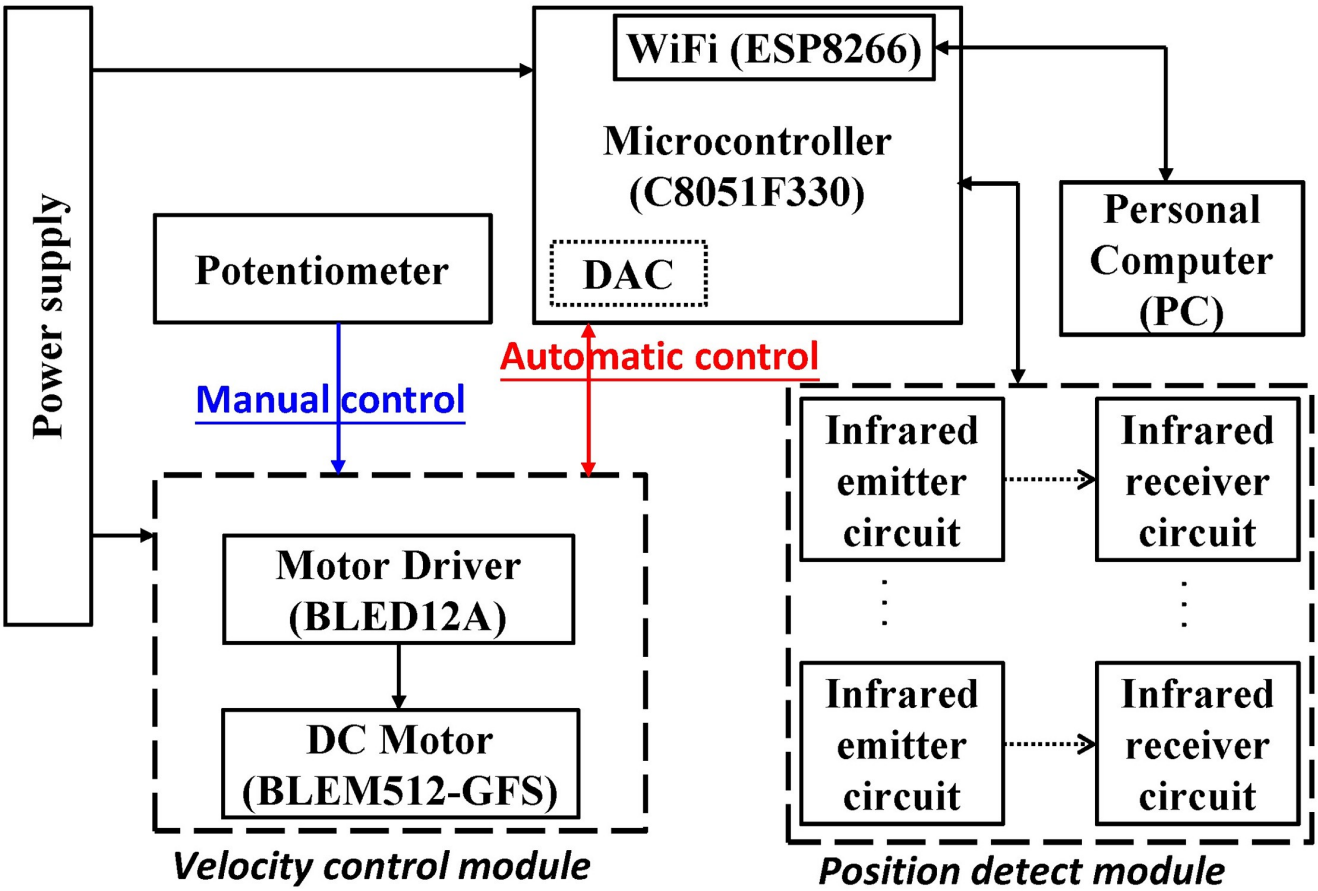
Links among the manual/automatic controllers and a position detection module.

As illustrated in Fig 3, the controller hardware was designed to have a manual control mode and an automatic one. In the former mode, an ORIENTAL BLEM512-GFS DC brushless motor (TAIWAN ORIENTAL MOTOR CO., LTD.) was driven by the output of a manual potentiometer through a BLED12A motor driver, while a Silicon Labs C8051F330 microcontroller (MCU) with a built-in DAC and IR transmitter/receiver pairs was employed for the purpose of position detection and motor speed control in the latter mode. Since rats were measured to have an average length of 22 cm, and the escalator is 110 cm long, six IR pairs were deployed along the escalator with a pitch of 22 cm, such that merely a single IR sensor was triggered at a time.

### Programmable running speed control

**Fig 4:**
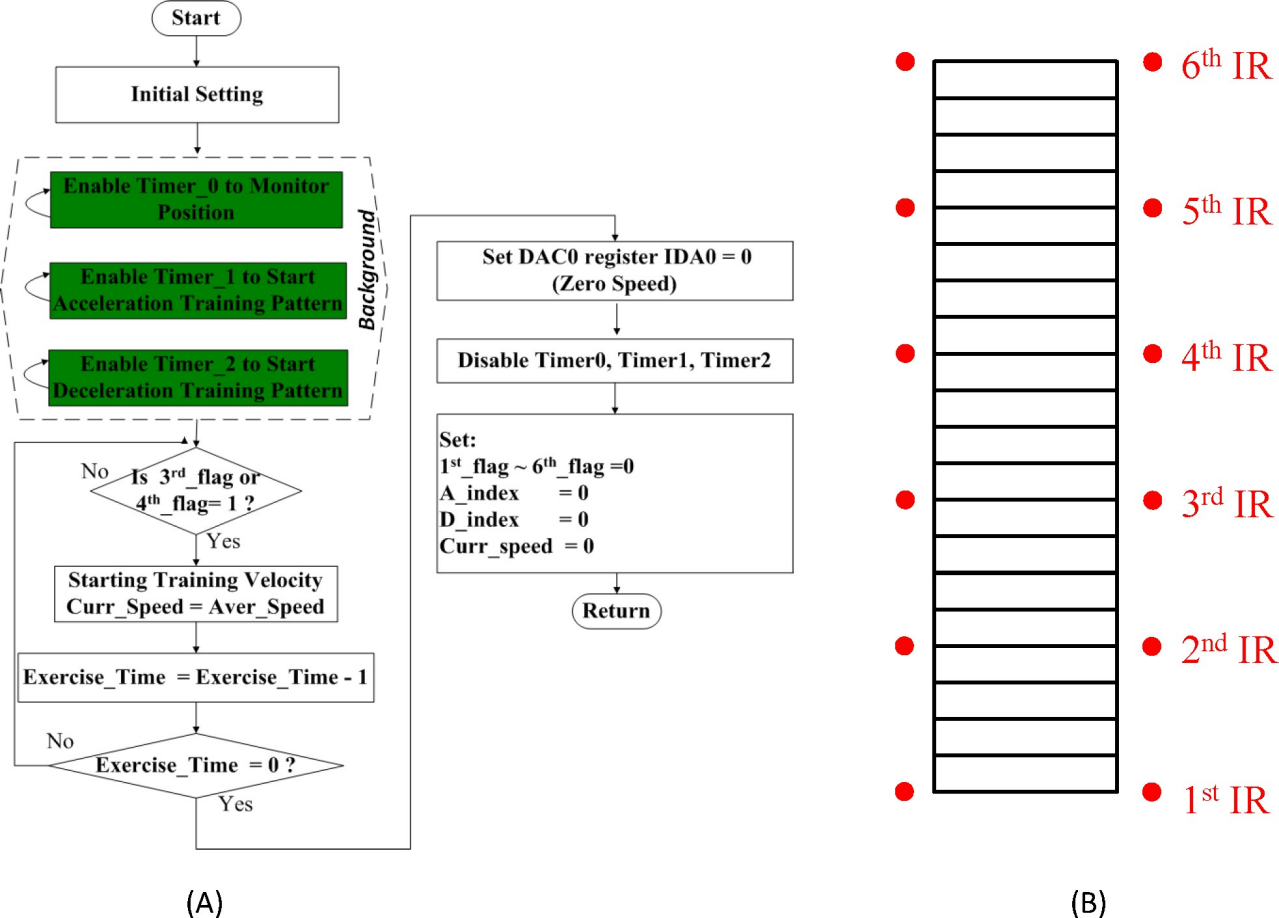
(A) Flow chart of the presented adaptive training program, (B) schematic representation of the 6 deployed IR transmitter-receiver pairs.

As in the hardware, there were a manual mode and an automatic mode in the controller software. To begin with, the most appropriate training strength was observed for rats in the manual mode, based on which a safe rehabilitation environment is provided in the automatic mode. As illustrated in Fig 4B, a total of 6 IR pairs were deployed and numbered upward, and three timer interrupt service routines were enabled by turns after initialization for position monitoring, acceleration and deceleration training, respectively, as shown in Fig 4A. Once IR receiver 3 was triggered by a rat, the feature “Starting Training Velocity” was enabled. The training speed was set to the average of the training speed for rats, covering a wide range of physical diversity, in need of rehabilitation. A 10-bit digital-to-analog converter (DAC) was enabled by a pulse-width modulation (PWM) signal, such that the motor speed could be controlled. In operation, the position of the rat was monitored, and the running speed of the escalator was tuned on a real-time basis, as highlighted in green in Fig 4A. Once IR receiver 1 was triggered again, it meant that the rat is unable to keep pace with the running speed, and the escalator was shut down instantly due to safety concerns. In contrast, once receiver 6 was triggered, it meant that the rat had reached the end of the escalator, and the escalator was powered off right away, just as in the previous case. Subsequently, the main program checked whether receiver 3 was triggered or not, while awaiting another run.

**Fig 5:**
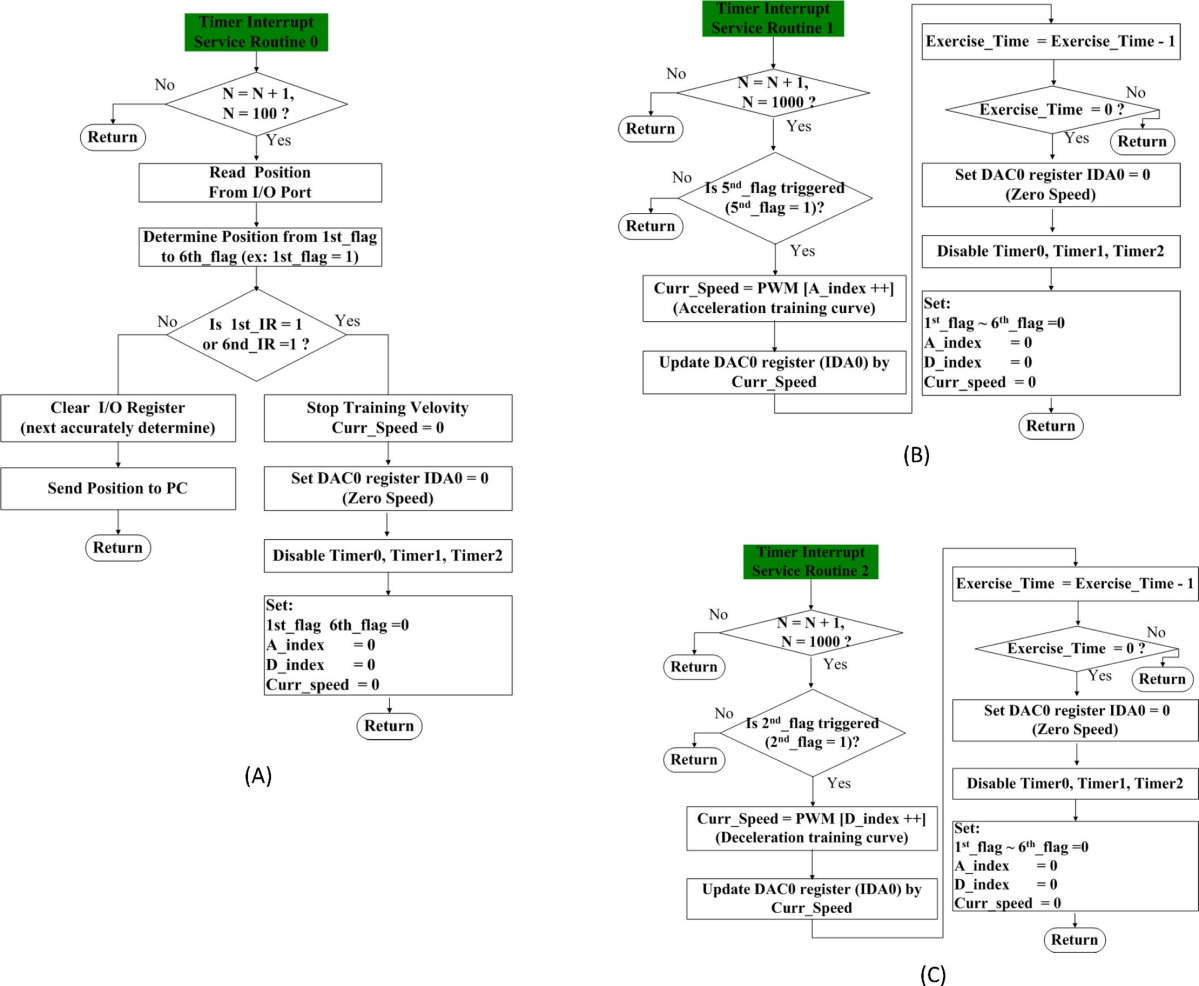
Flow chart of (A) timer interrupt service routine 0 for position detection, (B) routine 1 for acceleration, and (C) routine 2 for deceleration.

As illustrated in Fig 5A–5C, 3 timer interrupt service routines were designed to monitor the rat’s position and conduct acceleration/deceleration mechanisms, respectively. In Fig 5A, the timer interrupt service routine 0 shows how the MCU monitored the rat’s position via a general purpose I/O port by reading the 6-bit content of a P1 register every 0.1 s; the position information is recorded in a computer, and the content is reset for the next run. As was explicitly referred to previously, the escalator was shut down as a safety measure to prevent workout injuries in rats once either receiver 1 or 6 was triggered. An acceleration mechanism, illustrated in Fig 5B, was enabled in Timer interrupt service routine 1 for the operation of the escalator once the rat climbed the escalator faster than expected, and receiver 5 was triggered accordingly. The escalator accelerated at a constant rate in consistency with that observed in the manual mode. In contrast, a deceleration mechanism was conducted in Timer interrupt service routine 2, as illustrated in Fig 5C; once receiver 2 is triggered, the rat had moved upward at a slower-than-expected pace. As in the acceleration case, the deceleration value is determined through observation in manual mode. The escalator continued to decelerate until the rat reached the interval between receivers 3 and 4, and then the escalator resumed its operation at the initial running speed. As it turned out, a rat can be well and safely rehabilitated via a combined use of 3 timer interrupt service routines.

A graphical user interface (GUI) was written using the C++ programming language, and got connected to the PC and the microcontroller via an ESP8266 Wi-Fi module. When the PC took control of the MCU, parameters, including the training duration and the sensor response time, can be directly configured on the GUI. In addition, the moment the “start” button is clicked, the location and running speed of a rat were sensed and then saved as text files in the PC.

### System performance validation

As a preliminary step of the system performance validation, the location capturing function was tested in both the manual and automatic modes. In manual mode, each IR receiver was triggered using a piece of cardboard to block the received beam, and checked whether the triggered signal was well delivered to the MCU. In automatic mode, the cardboard was attached to the escalator with a running speed ranging between 0.5 and 10 m/min, and the location capturing function was validated as 100% reliable, as in the manual mode.

### Animal tests

In this work, rats were rehabilitated using the built rat escalator. Middle cerebral artery occlusion (MCAo) surgery was performed on the rats, and a 3-week rehabilitation program commenced a week later.

#### Grouping

The experimental animals were male Sprague-Dawley rats weighing approximately between 270–320 g, and were provided by BioLASCO, Taiwan. The rats were maintained in an air-conditioned animal chamber, and the chamber temperature was set at 26 ± 0.5°C. A light/dark cycle of 12 hours was used, and unlimited water and feed were provided. All of the experiments were conducted in daytime conditions under light. The experimental procedures were approved by the Animal Ethics Committee of the Chi Mei Medical Center (approved IACUC no. 105122607), Taiwan.

All of the rats were randomly divided into the sham, control, treadmill and AE groups. MCAo surgeries were performed on all the group members, excluding those in the sham group, and both the sham and control group members did not take part in the rehabilitation program afterwards. Motor recovery was assessed using an incline plane test and a rotarod test. All of the rats were then sacrificed right after the rehabilitation program, and the infarction volume was measured using the triphenyltetrazolium chloride (TTC) staining method [34].

#### Middle cerebral artery occlusion (MCAo)

The induction procedure of an MCAo surgery was based on Longa’s brain endovascular occlusion method [35]. All animals were anesthetized with 40 mg/kg Zoletil and 10 mg/kg xylazine via intraperitoneal injection. After the rats were anesthetized, a midline incision was made 0.1 cm left of the midline of the neck to expose and then tie the left common carotid artery (CCA) using a white suture, until the middle cerebral artery (MCA) was blocked to induce a cerebral ischemic stroke. Sixty minutes later, reperfusion was achieved by withdrawal of the suture, and the wound was sutured.

#### Rehabilitation program

**Fig 6:**
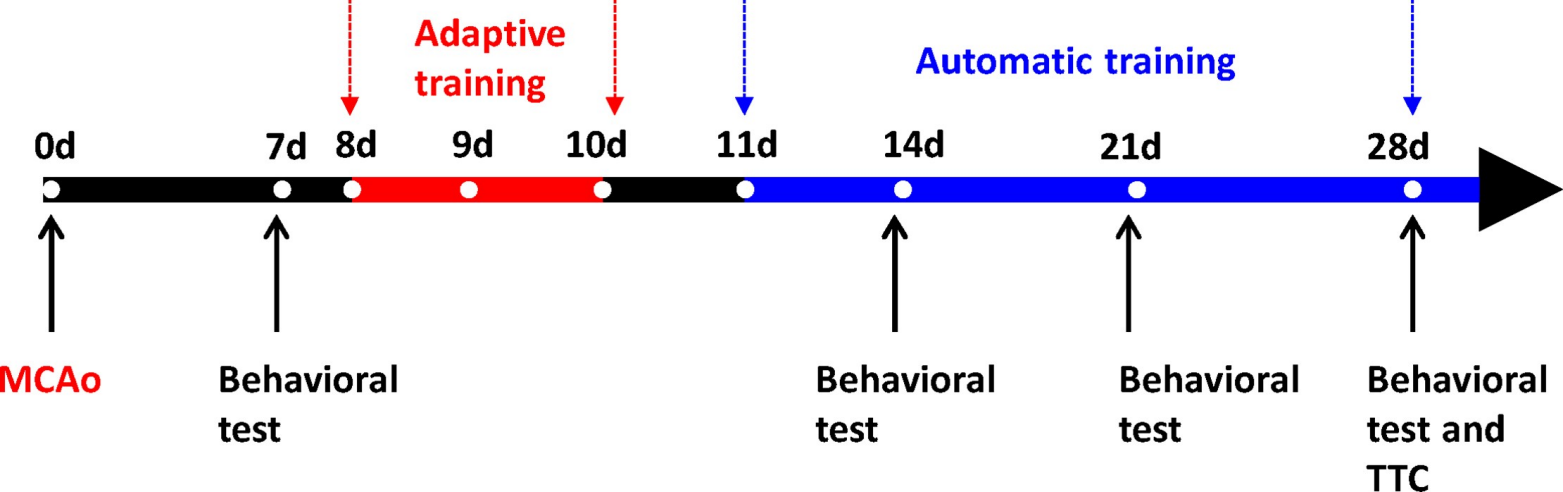
Timeline for the rehabilitation performance validation.

As illustrated in Fig 6, there is an adaptive training program followed by an automatic program on the timeline. It was stated explicitly and previously that MCAo surgeries were performed on rats a week before the 3-day adaptive training program. On Day 1 of the program, the rats were placed on the escalator, got accustomed to the rehabilitation environment, and then climbed the ladder of their own free will. On Day 2, the running speed of the escalator was tuned manually, such that the rats could be trained in a midway position, the interval between IR sensors 3 and 4, as much as possible. When a rat climbed faster than expected, IR receiver 5 was triggered, and the escalator accelerated, and vice versa. A database was constructed containing the training speed, escalator acceleration and deceleration for the subsequent automatic training program. It took the rats an average of 30.4 s to climb from the bottom to the top of the 110 cm long escalator when not in operation. In other words, the rats climbed at an average speed of 2.1 m/min, as a reference to fine tune the running speed of the escalator in the automatic training program. This fine-tuning procedure was repeated until the optimized running speed, acceleration and deceleration were obtained in terms of regular training purposes. The training duration and the running speed at the finish line were recorded for each rat. In this work, the escalator was designed to have an average running speed of 2.4 m/min, at which IR receivers 3 and 4 were triggered to accelerate at a constant rate of 31.2 m/min^2^ until receiver 5 is triggered. A speed of 5 m/min was reached at t = 5 s and then the escalator decelerated at 42 m/min^2^ after receiver 2 was triggered, until 1 m/min was reached at t = 2 s. The running speeds during acceleration and deceleration were respectively expressed as

V(t)=0.54t+2.4,0≤t≤5s (1)

V(t)=−0.7t+2.4,0≤t≤2s (2)

The rehabilitated group was further divided into the treadmill and AE subgroups. As presented in [9, 25], the treadmill subgroup members underwent a 3-week training program, i.e., an exercise duration of 30 min at a speed of 15 m/min on a daily basis over weeks 1 and 2, and 60 min at 20 m/min over week 3. For comparison purposes, the AE training program, illustrated in Fig 6, offered the same exercise duration as those in [9, 25].

#### Behavior tests

After the MACo surgeries, motor function recovery was assessed on a weekly basis using an incline test and a rotarod test, respectively. In the former test, the rear limbs of a rat were placed inside a black box carpeted with a rubber ribbed surface on the upper end of an inclined plane, while the fore limbs were on the exterior of the box. Starting at 25°, the inclination angle continued to grow until the rat slid downward. The angle was recorded as a quantitative measurement of the hind limb grip strength, and the test terminated right away [36]. In the latter test, the rat’s sense of balance and motor coordination function were quantified using a Panlab LE 8505 Accelerating Rota Rod. The 30 cm high rod, carrying up to 4 rats at a time, speeded up from an initial rotational speed of 4 rpm to 30 rpm at t = 4 min, and reached a final speed of 30 rpm within a maximum duration of 5 min. The test terminated once the rat fell to a load cell below due to fatigue or loss of balance, and the exercise duration and the rotational speed were recorded instantly and automatically. As in [37], the test was conducted on each rat triple times per day with a 2-min break between trials.

#### Cortisol assay

Blood samples were obtained from the tail vein of the animals in both the Treadmill and the AE groups before stroke and on Days 14, 21 and 28 for measuring the cortisol levels. Rats were placed in a plastic restraining holder, and blood samples (1 ml/rat) were collected into the vacuum blood collection tube at each instant from the tail vein. Centrifuge the blood sample collection tubes in a refrigerated centrifuge at 3,000 x g for 10 min to separate serum. Separated serum was collected and stored at −80°C until further analysis. The serum concentrations of cortisol were measured by enzyme-linked immunosorbent assay using a commercial kit (Kit Cat. # ADI-900-071, Enzo Life Sciences, Farmingdale, NY, USA), following the manufacturer’s instructions. The samples were assayed in triplicate, and the values were expressed as means ± S.D.

#### Nissl and MAP2 staining

After the last blood sampling on Day 28, rats were euthanized with sodium pentobarbital (120 mg/kg, i.p.; Sigma-Aldrich, St. Louis, MO, USA) and intracardially perfused with 4% paraformaldehyde in phosphate-buffered saline (Sigma-Aldrich). Following perfusion, the brains were removed and immersed in the 4% paraformaldehyde overnight, and then were embedded in paraffin. Ten series of 10-μm-thick coronal sections were cut every 100 μm from the covered injured cortex and the striatum. Five coronal sections were incubated in a solution of 0.1% cresyl violet (Sigma-Aldrich) to measure the lesion volume in the ipsilateral hemisphere. The lesion volume was calculated as previously described [38] using software (Image-Pro Plus 6.0, Media Cybernetics Inc., Bethesda, MD, USA), and the relative lesion volume was expressed as a percentage of the contralateral hemisphere. Another five coronal sections were incubated with microtubule‐associated protein 2 (MAP2; Abcam, Cambridge, UK) for neuron dendrite [39]. Cell counting was performed on four randomly selected nonoverlapping fields in the ipsilateral and contralateral of cortex and striatum regions per section by an independent observer blinded to the experimental groups. The number of MAP2‐positive neurons was determined using ImageJ software, 1.49v (National Institutes of Health, Bethesda, MD), and expressed as a percentage of the contralateral regions.

### Performance statistics

The mean values of the test results were presented together with the standard deviations (SD). The performance was analyzed using a repeated-measure ANOVA, and the performance difference between the two groups was evaluated using a t-test. P < 0.05 was considered a significant performance difference between groups.

**Fig 7:**
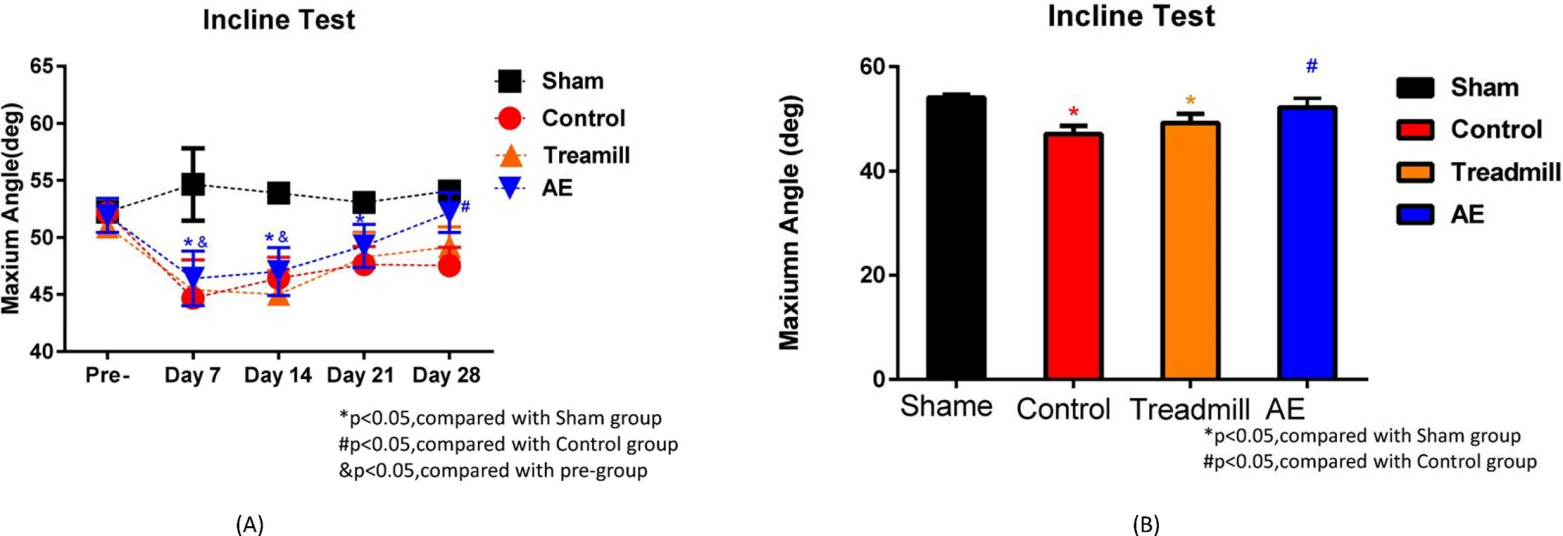
(A) Comparison of the incline plane test results on a weekly basis and (B) comparison of the results on Day 28.

**Fig 8:**
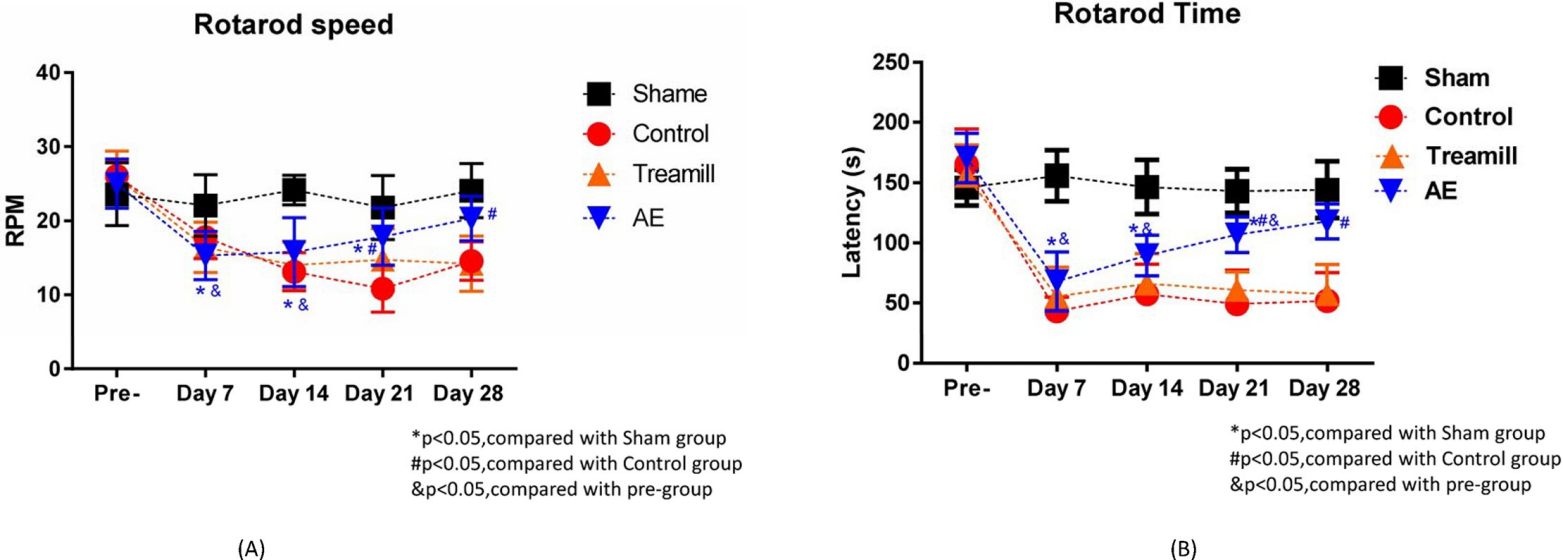
Comparison of (A) the rotational speed and (B) the latency of the rotarod test on a weekly basis.

**Fig 9:**
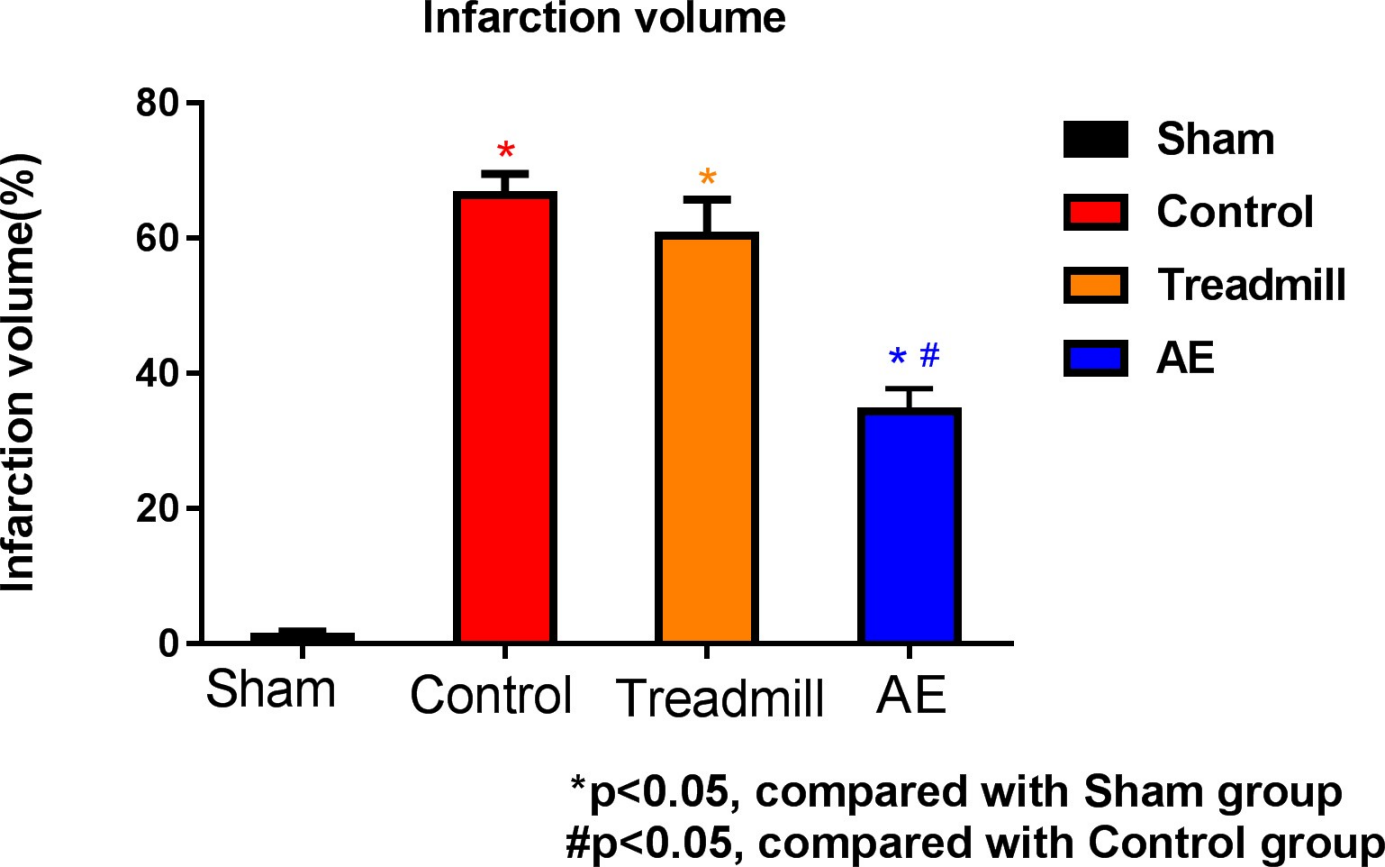
Comparison on the average infarct volume on Day 28 using the TTC staining method.

**Fig 10:**
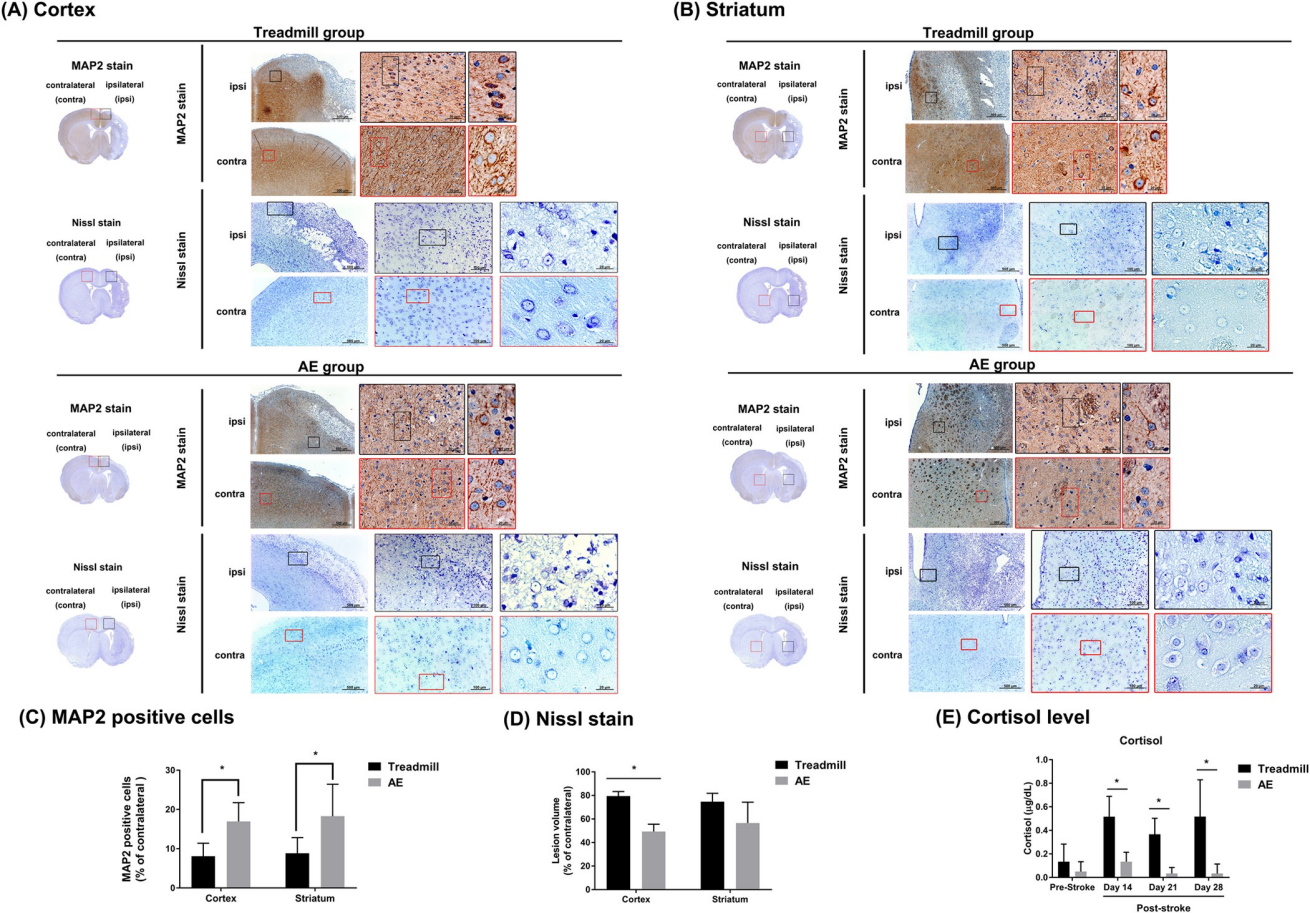
Nissl and MAP2-stained (A) cortical and (B) striatal tissues of a rat in the treadmill and the AE groups following the MCAo surgeries. The framed image in a full-size MAP2-stained and Nissl-stained brain image is zoomed in as the full-sized image on the right. Comparison on (C) the number of the MAP2-stained cells, (D) the lesion volume and (E) the level of cortisol in the cortex and the striatum between groups. Data are presented as means ± SEM (n = 5, *p < 0.05). Please note that neither the AE nor the treadmill group in Fig 10 contains the same members as the counterparts in Figs 7–9. This is simply because the tests and the infarct volume measurements in Figs 7–9 were carried out approximately a year earlier than those in Fig 10. In other words, they are *physically different*, while can be considered *statistically identical* in terms of rehabilitation performance.

As will be seen right below, Figs 7–9 give incline plane test, rotarod test results and the average infarct volume, and Fig 10 gives the number of MAP2 positive cells, the lesion volume and the level of cortisol. However, it must be pointed out that neither the AE nor the treadmill group in Fig 10 contains the same members as the counterparts in Figs 7–9. This is simply because the tests and the infarct volume measurements in Figs 7–9 were carried out approximately a year earlier than those in Fig 10. In other words, they are *physically different*, while can be considered *statistically identical* in terms of rehabilitation performance.

## Results

### Incline plane

After the MCAo surgeries, the rear limb grip strength of the stroke rats was assessed on a weekly basis over a 28-day time period. Members of both the treadmill and AE groups underwent a 3-week training program, as illustrated in Fig 6, a week after the MCAo surgeries. Fig 7A gave a weekly comparison on the incline test performance among groups, while the comparison results on Day 28 was exclusively exhibited in Fig 7B. As presumed, the sham group was found in Fig 7A to outperform the others, particularly the control group, through the observation period, but excluding the pre-training. Furthermore, there was no significant difference in the test performances on Day 21 and the pre-training for the AE group, while a significant performance difference between the AE and control groups, as shown in Fig 7B. Nevertheless, little performance differences were seen between the control and treadmill groups. Hence, AE was experimentally validated as effective in terms of grip function recovery for rats with ischemic stroke.

### Rotarod

Fig 8A showed the rotarod counterpart of Fig 7A in terms of the rotational speed, while Fig 8B gave a comparison on the average latency, measured in s, among groups over the 28-day observation period. As can be seen in Fig 8A, there was an obvious significant difference between the AE and control groups on Days 21 and 28, i.e., P < 0.05. This observation applies to the rotarod test results in Fig 8B (P < 0.05). As in the incline test case, the AE group was found to have a comparable performance as compared with the control group throughout the entire observation period. In short, AE was experimentally validated as effective in terms of sense of balance and motor coordination recovery.

### Infarct volume

Using the TTC staining method, normal cells appear red, while dead cells are highlighted in white instead, and the infarction volume can be measured accordingly. As can be seen in Fig 9, the treadmill group gave an infarction volume of (60.36 ± 5.3)%, and the control counterpart had (66.41 ± 3.1)%, meaning that there was an indistinguishable statistical difference between both in this regard. Nonetheless, the AE group had an infarction volume as low as (34.36 ± 3.3)%, a significant outperformance over the control group, i.e., P < 0.05. Hence, AE was experimentally validated as superior in this regard for the rehabilitation of rats.

Fig 10A gives Nissl and MAP2-stained tissues in the cortex of a rat in the AE and the treadmill groups, while Fig 10B gives those in the striatum following the MCAo surgeries. The framed image in a full-size MAP2-stained and Nissl-stained brain image is zoomed in as the full-sized image on the right. As will be seen below, the performance of a rehabilitation platform is further quantified as the number of MAP2 positive cells and the lesion volume, and the mental stress is as the cortisol level for comparison purposes.

### MAP2 positive cells

Immunohistochemical methods were performed to detect the number of surviving neurons, also referred to as Microtubule-associated protein 2 (MAP2) positive cells, following MCAo surgeries. MAP2 is a cytoskeletal marker mainly localized to dendrites of neurons in the mature rat brain [40]. As can be seen in Fig 10C, the AE group statistically outperformed the treadmill counterpart with respect to the number of MAP2 positive cells, that is, (16.97 ± 4.8)% vs. (8.08 ± 3.3)%, in the cortex case. In the striatum case, the AE group outperformed the treadmill counterpart again, that is, (18.29 ± 8.1)% vs. (8.84 ± 3.9)%. Besides, the result, provided by the AE group, in the striatum case turned out to be comparable with that in the cortex case, that is, (18.29 ± 8.1)% vs. (16.97 ± 4.8)%.

### Lesion volume

Nissl staining was performed to analyze gross anatomical structure changes [41], and damaged brain tissue was evident via a loss of Nissl intensity with the pyknotic and apoptotic neurons [42]. In Nissl stained-tissues, true degenerating neurons appear pale because of the dissociation of ribosomes from the rough endoplasmic reticulum [43]. Percent lesion volume was defined as each ipsilateral lesion volume divided by the total contralateral hemisphere volume. Lesion volumes were measured using the Nissl staining, and then compared in Fig 10D. As can be seen therein, the AE group outperformed the treadmill counterpart, that is, (27.77 ± 2.8)% vs. (41.02 ± 0.8)%, in the cortex case, in agreement with the infarct volume case. In the striatum case, the AE group appeared to outperform the treadmill counterpart again, that is, (8.16 ± 2.8)% vs. (17.44 ± 2.7)%, while unfortunately there was no statistical difference between both groups this time.

### Cortisol levels

Cortisol levels were measured, and then compared in Fig 10E. As presumed, there was no statistical difference between the treadmill and the AE groups (0.13 ± 0.1 vs. 0.05 ± 0.08) before MCAo surgeries. Instead, a statistical difference was observed on Day 14, the 7th day of the 3-week rehabilitation program, between both groups (0.52 ± 0.2 vs. 0.13 ± 0.08). On Day 21, the AE group gave a cortisol level of 0.03 ± 0.05, a figure far lower than that on Day 14, while the treadmill counterpart gave 0.37 ± 0.1. On Day 28, the AE group gave 0.03 ± 0.08, statistically identical to the Day 21 case, while the treadmill counterpart gave 0.52 ± 0.3. It is noted that the AE group gave consistent and extremely low levels of cortisol throughout the entire observation period, excluding Day 14. This finding solidifies the argument that rats can be well rehabilitated under mental stress-free circumstance using an adaptive platform.

## Discussion

In this paper, rats can be well rehabilitated using an adaptive escalator. Taking into account the physical diversity among patients, the running speed, i.e. the training strength, of the escalator was made adaptive as a reliable way to completely eliminate the workout injuries suffered in a conventional treadmill. Furthermore, commercial animal training platforms, as those in [7, 8], have been validated as effective in terms of stroke prevention, but not for rehabilitation purposes [9–11]. This may be due to the fact that a stroke rat is definitely physically weaker than a healthy one. For this reason, overtraining rats may lead to workout injuries, e.g. fall injuries once they cannot keep pace with treadmills, or mental stress due to an imposed electric shock. As highlighted in [9, 10], a rehabilitated group may even underperform a control counterpart by undergoing a poorly designed rehabilitation program, the worst-case scenario. Accordingly, an adaptive training mechanism was developed herein as a solution to the above-referred issues.

Finger and toe movements had been experimentally validated to stimulate respective parts of stroke patients’ brains [44]. More precisely, it is that the movements activate the frontal lobe and corticofugal fibers. From our point of view, this finding could be generalized to rat cases, although there is nothing available yet in the literature to support this argument. In other words, this issue has not been well argued here, and more evidence is required to solidify this argument.

Conceptually, a ladder-climbing movement uses more muscle than required when simply jogging. Simply speaking, it is the finger and toe movements when grabbing the railway of the escalator under mental stress-free conditions herein, and respective parts of the impaired brain are presumed to be stimulated and even revitalized accordingly, as argued earlier. This ladder climbing feature gives the escalator-based rehabilitation mechanism a clear advantage over a number of existing counterparts. Table 1 shows a feature comparison among a treadmill, a robot-assisted counterpart [12] and this approach.

**Table 1:** Feature comparison among rehabilitation platforms.

Features	Treadmill [9]	Robot-assisted platform [12]	This proposal
Runway material	Long-shaped rubber belt	Long-shaped rubber belt	Railways
Training model	Forced (electrically stimulated)	Forced (suspended and guided by a robotic arm)	Forced (motor-driven wheel)
Type of rehabilitation	Jog	Walk	Climb
Adaptive speed control	-	+	+
No. of rats trained at a time	Multiple	Single	Single
Position detection	-	-	+
Fall prevention	-	+	+
Automatic acceleration training	-	-	+
Automatic deceleration training	-	-	+
Injury prevention	-	+	+
Mental stress level	High	Low	How
Cost	High	High	Moderate

Note that “+” and “-” identify available and not available, respectively.

As illustrated in Figs 7–9, the AE group was found to outperform the control group in terms of motor function recovery over the entire observation period, particularly the infarction volume after sacrifice (P < 0.05). The presented training mechanism has a major advantage over commercial training platforms, since the training strength was made adaptive, taking account of the physical diversity among rats, and this rehabilitation environment is completely workout injury-free accordingly. Hence, the presented adaptive training platform was validated as effective for rehabilitation purposes.

Besides, the average performance of all the rehabilitated rats was found to improve week by week, even though two rats underperformed the others. This is definitely an advantage over commercially forced training platforms.

In addition to the infarct volume comparison, lesion volumes and the number of MAP2 positive cells were measured using the Nissl staining and immunohistochemical methods, respectively, and then compared in the cortex and the striatum cases. The AE group turned out to statistically outperform the treadmill counterpart with respect to the number of MAP2 positive cells in both cases, while with respect to the lesion volume only in the cortex case. This is simply because there was no statistical difference in the lesion volume between the AE and the treadmill groups in the striatum case.

The mental stress of electrically stimulated rats on a treadmill has a negative effect on a convincing interpretation of collected physiological data. Ideally, rats should be trained on a stress- and injury-free training platform. In light of this, this paper presented an adaptive training mechanism, implemented in an escalator and taking into account the physical diversity among rats. As many know, mental stress can be quantified as the level of cortisol. The levels of cortisol, experienced by AE group, were found to be much lower than those by the treadmill counterpart, and were even comparable to the levels before MCAo surgeries in some cases. This finding well solidifies the argument that rats can be rehabilitated under mental stress-free circumstance using an adaptive rehabilitation mechanism. The outperformance of this mechanism was validated by experimental means, and this adaptive rehabilitation mechanism can be applied to physiology-related studies in the future.

## Conclusion

In this work, rats with ischemic stroke were well rehabilitated using an escalator. Considering the physical diversity among rats, the escalator was built in such a way that training strength was made adaptive. Accordingly, this training mechanism was experimentally validated to outperform a conventional treadmill, particularly with respect to the brain infarct volume, the lesion volume, the number of MAP2 positive cells and the level of cortisol. Presumably, this is due to the fact that ladder-climbing movement uses more muscles than it takes to simply go jogging. In addition, this escalator, unlike a forced treadmill training platform, was designed as an adaptive and mentally stress-free rehabilitation environment. So far, major inherent disadvantages, such as mental stress and secondary injury, of animal training platforms are believed to result in non-convincing arguments. Hopefully, experiments in basic and clinical research can lead to more convincing arguments using this work.
